# Stereopsidales - A New Order of Mushroom-Forming Fungi

**DOI:** 10.1371/journal.pone.0095227

**Published:** 2014-04-28

**Authors:** Elisabet Sjökvist, Bernard E. Pfeil, Ellen Larsson, Karl-Henrik Larsson

**Affiliations:** 1 Department of Biological and Environmental Sciences, University of Gothenburg, Gothenburg, Sweden; 2 Natural History Museum, University of Oslo, Oslo, Norway; University of Minnesota, United States of America

## Abstract

One new order, one new family, and one new combination are presented, as the result of molecular phylogenetic analyses. The new order Stereopsidales and the new family Stereopsidaceae are described incorporating *Stereopsis radicans* and *S. globosa*, formerly *Clavulicium globosum*. We show that not only do these species represent an old overlooked lineage, but both species harbor cryptic diversity. In addition, a third species, *C. macounii*, appears as a plausible sister to the new lineage, but there is conflict in the data. All specimens of *S. radicans* and *S. globosa* analysed here are from the South and Central Americas; several records of *S. radicans* have been made also from tropical Asia. We expect the true diversity in this group to be a lot higher than presented in this paper. *Stereopsis radicans* was formerly included in Polyporales, but a placement within that order is rejected by our data through SH tests. The dataset consisted of four nuclear markers: *rpb2*, *tef1*, LSU and SSU, each of which was analysed separately using maximum likelihood and Bayesian inference. Recombination detection tests indicate no plausible recombinations. The potential of *S. radicans*, *S. globosa* and *C. macounii* being amphitallic is briefly discussed.

## Introduction

Agaricomycetes Dowell, commonly recognized as the mushroom forming fungi, are basidiomycetous fungi forming complex fruiting bodies above and below ground. At present, the Agaricomycetes includes 19 orders [1], [2] dominated by lineages with agaricoid, corticioid, boletoid, polyporoid, and gasteroid sporocarps [3]–[6]. The presence of another unrecognized order within Agaricomycetes was indicated in a recent study of stipitate stereoid fungi, based on nuclear ribosomal 5.8 S and 25 S (LSU) data [7]. Most stipitate stereoid taxa could at that time be assigned to either of the orders Polyporales Gum., Hymenochaetales Oberw., Atheliales Jlich, or Agaricales Underw. However, *Stereopis radicans* (Berk.) D. A. Reid, the type of *Stereopsis* D.A. Reid, together with *Clavulicium globosum* Hjortstam & Ryvarden formed a sister clade to the Cantharellales Gum., the Phallomycetidae Hosaka, Castellano & Spatafora, and remaining orders of Agaricomycetes. *Clavulicium macounii* (Burt) Parmasto, the type species of *Clavulicium* Boidin, also did not appear as a member of any of the recognized orders, but separate from the *S. radicans* - *C. globosum* clade.

Here we investigate the robustness of the *Stereopsis radicans* - *Clavulicium globosum* clade and the placement of *C. macounii* by analysing the nuclear markers RNA polymerase II subunit (*rpb2*), translation elongation factor 1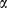 (*tef1*), and nuclear small subunit ribosomal (SSU) DNA as well as LSU. In addition to phylogenetic analyses, the strength of the Maximum Likelihood trees are tested against alternative trees that would necessitate new taxonomic combinations or that would support existing morphological classifications. The potential of the study group being of a hybrid origin or having received lateral gene transfer leading to disparate phylogenetic signals is investigated through recombination detection tests. These tests would also reveal if there were any chimeric sequences. Gene tree incongruencies are common in the studies of plants and animals [8], [9], but less well investigated in fungal phylogenetics. We are open to the possibility that the genetic markers studied here have different evolutionary histories, and therefore choose to analyse each gene separately.

We show that a new order is required to adequately summarize the unique evolutionary history represented by these fungi. We also delimit the group of study in relation to the current classifications of Hibbett et al. and Binder et al. [1], [2], and in doing so, show that this newly described order has a previously unrecognized ancient history stretching back 237–290 million years based on comparisons with dated phylogenies of the Agaricomycetes [10].

## Results

### Phylogenetic analyses

**Figure 1:**
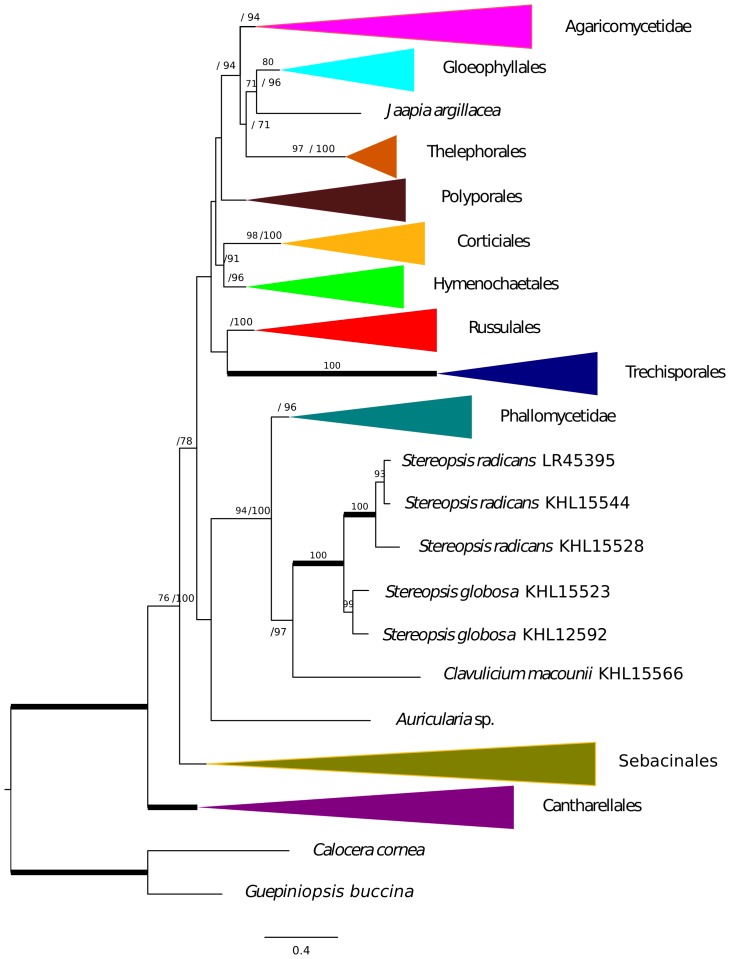
*Rpb2* phylogeny. Maximum likelihood tree of *rpb2* with Bootstrap/Bayesian frequencies as a percentage shown above branches. Thick branches receive full support of both Bayesian frequencies and Bootstrap. The collapsed and colored groups represent current orders and subclasses of the Agaricomycetes.

**Figure 2:**
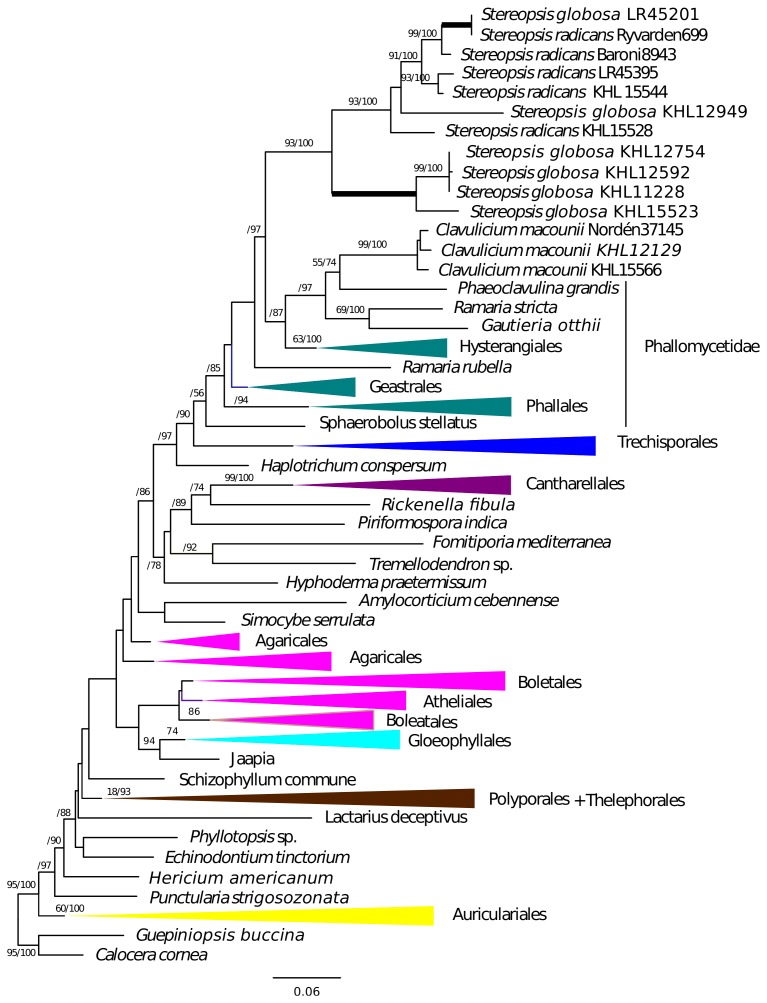
*Tef1* phylogeny. Maximum likelihood tree of *tef1* with Bootstrap/Bayesian frequencies as a percentage shown above branches. Thick branches receive full support of both Bayesian frequencies and Bootstrap. The collapsed and colored groups represent current orders and subclasses of the Agaricomycetes.

**Figure 3:**
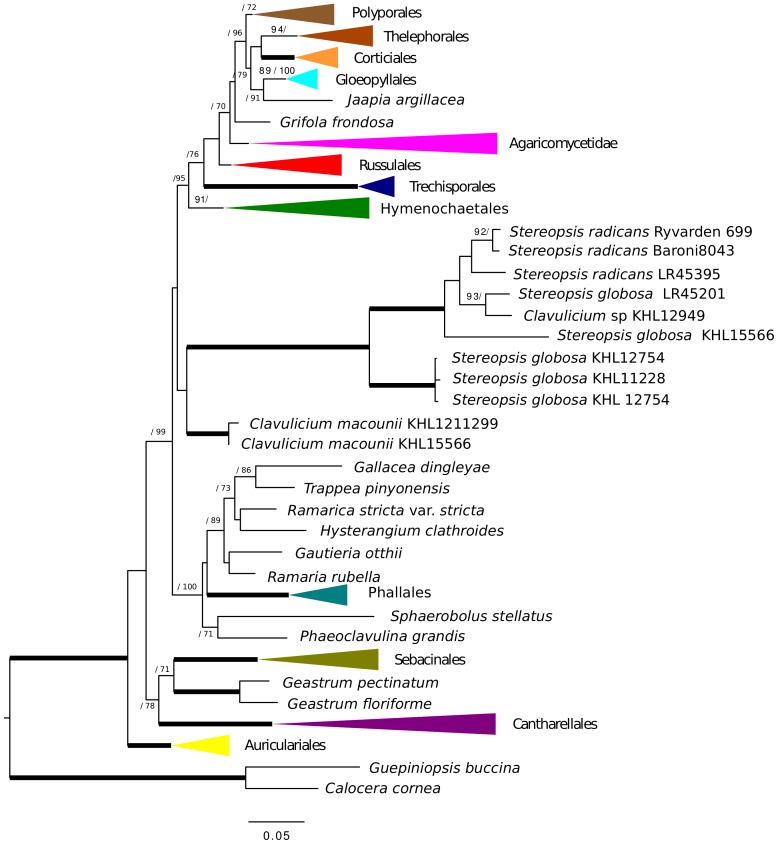
LSU phylogeny. Maximum likelihood tree of nLSU with Bootstrap/Bayesian frequencies as a percentage shown above branches. Thick branches receive full support of both Bayesian frequencies and Bootstrap. The collapsed and colored groups represent current orders and subclasses of the Agaricomycetes.

**Figure 4:**
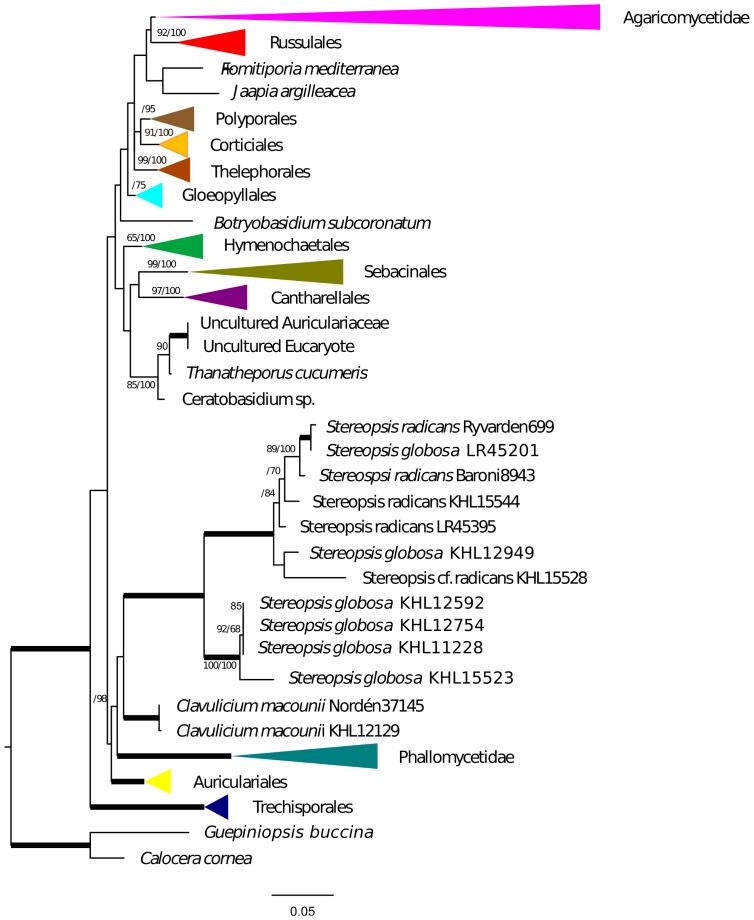
SSU phylogeny. Maximum likelihood tree of nSSU with Bootstrap/Bayesian frequencies as a percentage shown above branches. Thick branches receive full support of both Bayesian frequencies and Bootstrap. The collapsed and colored groups represent current orders and subclasses of the Agaricomycetes.

Gene tree analyses of *tef1*, *rpb2*, SSU and LSU all reveal the same relationships with regards to *Stereopsis globosa* and *S. radicans*, namely that these species form a clade of their own in each tree. The branches supporting the monophyly of *S. radicans* and *C. globosum* received 93% bootstrap or higher and a Bayesian posterior probability of 1.0 (Figs. 1, 2, 3, 4). All trees are available from http://purl.org/phylo/treebase/phylows/study/TB2:S14833 for download. In contrast, the placement of *C. macounii* is not equally clear. Samples from this species appear as the sister group to the *S. radicans* - *C. globosum* clade in analyses of *rpb2*, SSU and LSU, but with bootstrap supports only up to 63% and posterior probabilities between 0.53 and 0.97. *Clavulicium macounii* was not found to be sister to *S. radicans* - *C. globosum* in analyses of *tef1*. Analyses of *rpb2* and SSU reveal a sister relationship between Phallomycetidae and the *S. radicans* – *C. globosum* – *C. macounii* clade, whereas the phylogeny of *tef1* shows *C. macounii*, and the *S. radicans* - *C. globosum* clade as a part of a paraphyletic Phallomycetidae. The gene tree of LSU shows the Phallales as polyphyletic, but not as sister to the *S. radicans* – *C. globosum* – *C. macounii* clade.

The *Stereopsis radicans* - *S. globosa* clade split into two well separated lineages, one containing sequences only from *S. globosa* specimens, whereas the other contains sequences from both *S. globosa* and *S. radicans* specimens. Three specimens of *S. globosa* have almost identical sequences for several markers, but the remaining specimens appear to be clearly differentiated (Figs. 1, 2, 3, 4).

### Additional analyses

We tested whether alternative groupings could be rejected by our data using Shimodaira-Hasegawa (SH) topology tests [11]. The SH tests of monophyly constraints rejected a monophyletic group consisting of Polyporales Gum., *Stereopsis radicans*, *Clavulicium globosum* and *C. macounii* for all markers (Table 1). A monophyletic clade consisting of *C. globosum*, *C. macounii* and *S. radicans* was present in three of the markers, and therefore not available to be tested as an alternative. However, in *tef1*, this alternative grouping was available and was rejected by the SH test (Table 1). The monophyly of *C. macounii* with the Phallomycetidae could not be ruled out by the SH tests of SSU, LSU and *rpb2*, but was rejected in the analyses of *tef1*. Analyses of LSU recovered *S. radicans* and *C. globosum* as sister to *C. macounii*, but this clade was not recovered as sister to the Phallomycetidae. However, the monophyly constraint of *S. radicans*, *C. globosum* and *C. macounii* together with the Phallomycetidae could not be rejected in the SH test performed on the LSU.

**Table 1:** Results of the SH tests.

		Marker		
Hypothesis	*rpb2*	*tef1*	SSU	LSU
H0	−62365.826866	−21287.959280	−16991.358317	−19069.923892
H1	-	Yes/−21505.315272	-	-
H2	-	Yes/−21510.178188	-	No/−19078.565209
H3	-	Yes/−21516.149702	-	No/−19079.216286
H4	No/−62373.423522	Yes/−21513.383437	No/−17019.475756	No/−19082.748004
H5	Yes/−62509.372069	Yes/−21427.239262	Yes/−17128.704432	Yes/−19210.024808

The question of whether the tested hypothesis results in a significantly worse tree than the ML tree under a p value of 0.05 is answered by a yes or a no. Log Likelihood values for the best tree under each hypothesis is given as a negative value. H1: *Stereopsis radicans*, *C. macounii*, *C. globosum* monophyletic. H2: Phallomycetidae, *Stereopsis radicans*, *C. macounii*, *C. globosum* monophyletic. H3: Phallomycetidae monophyletic and *Stereopsis radicans*, *C. macounii*, *C. globosum* monophyletic. H4: Phallomycetidae, *C. macounii* monophyletic H5: Polyporales, Stereopsidales monophyletic.

The best matches using blastn [12] searches with *Stereopsis radicans* and *Clavulicium macounii* as queries were inferred in all cases (except one) to be members of clades corresponding to the current orders. None of the best blastn matches appear as sister to any of the specimens of study in any of the gene trees.

The structure model analysis of SSU resulted in a similar tree to the regular analysis, and is therefore not shown.

Evidence for recombination events was assessed in RDP4, using the Geneconv, Chimaera, MaxChi, Secondary Bootscan and Secondary SiScan methods [13]–[17]. Detected recombination events were rechecked with all methods, but none of the detected recombinations appeared to be phylogenetically sound. None of the species of interest were recovered as recombinants when using a p-value cut off of 0.05.

## Discussion

*Stereopsis radicans* and *Clavulicium globosum* form a well supported clade, in all analyses performed here. This result is in concordance with previous analyses [7] based on LSU only. SH tests and ML trees refute the position of the *Stereopsis* lineage in Polyporales [18]. *Clavulicium macounii* appears to be sister to the *Stereopsis* clade, but this position is weakly supported, and rejected by the SH test in one marker. With the relatively short branch lengths supporting the sister relationship of the *S. radicans* - *C. globosum* clade and *C. macounii*, either position of *C. macounii* is plausible. The position of the *Stereopsis* lineage is separate from all currently recognized orders of Agaricomycetes Dowell, but appears as a sister lineage to *C. macounii* and the Phallomycetidae K. Hosaka, Castellano & Spatafora, this relationship is found in three markers. We do not have sufficient data to examine the rank of Phallomycetidae K. Hosaka, Castellano & Spatafora, a position within the subclass is possible but not convincing. A placement within any of the orders of Phallomycetidae K. Hosaka, Castellano & Spatafora is therefore also rejected.

We deem it necessary to describe a new order and a new family to incorporate the newly identified lineage. In doing so, we hope to draw more attention to this lineage which we believe is greatly under-studied, and we hope more records of the species will be reported. Corticioid species are not as well studied as other groups of Agaricomycetes, and the tropical countries, where this lineage is found, are still poorly sampled [19]. The alternative, to not make a formal classification, but instead introduce a temporary informal name, is less compelling. The lineage appears to be at least as robust as other higher ranked taxa in the Agaricomycetes. Our analyses also make it clear that it does not belong to any of the current orders. There is always uncertainty about how to demarcate ranked taxa, since there is no definitive delimiter. However, the ranks should reflect the unique history and predict the distinctive genetic diversity of a group. Therefore, increased knowledge of hitherto unrecognized ancient lineages and the naming and classification of such lineages into higher ranked taxa facilitates communication about them. Most importantly, such classifications also constitute highly important biodiversity information that goes beyond simply the number of species in a given place as the sole criterion for conservation.

### Taxonomy

*Stereopsis radicans* is the type of genus *Stereopsis*, and *Clavulicium macounii* the type of *Clavulicium*. We therefore deem it necessary to transfer *C. globosum* to *Stereopsis*, as *S. radicans* and *C. globosum* form a monophyletic clade and the monophyly with *C. macounii* is dubious. This would have no taxonomical impact should *C. macounii* later be proven sister to the *Stereopis* clade, so long as *S. radicans* and *C. globosum* remain monophyletic. However, we do recognize the possibility that there are several cryptic species that fit the description of the new combination, as well as within *S. radicans*.

***Stereopsis globosa*** **(Hjortstam & Ryvarden) Sjkvist comb. nov. Basionym;** ***Clavulicium globosum*** **Hjortstam & Ryvarden, Syn. Fung. (Oslo) 20∶35 (2005)** MycoBank number:MB805765.

**Stereopsidaceae Sjkvist, E. Larss., B.E. Pfeil & K. H. Larss., fam. nov.** MycoBank number:MB805764.

Type *Stereopsis* D.A. Reid, Nova Hedwigia, Beih. 18: 290 (1965). Homobasidiomycetes with effused, stipitate, spathulate or funnel shaped sporocarps. Hymenium smooth. Hyphal system monomitic, with clamps. Basidia clavate, exemplar species with two sterigmata. Cystidia present. Spores hyaline, smooth, upon drying becoming angular. In soil or on living or dead wood. Exemplar species: *Stereopsis radicans* (Berk.) D.A. Reid and *Stereopsis globosa* (Hjortstam & Ryvarden) Sjökvist.

**Stereopsidales Sjkvist, E. Larss., B.E. Pfeil & K. H. Larss., ord. nov.** MycoBank number:MB805763.

Type *Stereopsis* D.A. Reid, Nova Hedwigia, Beih. 18: 290 (1965) Homobasidiomycetes with effused, stipitate, spathulate or funnel shaped sporocarps. Hymenium smooth. Basidia clavate, exemplar species with two sterigmata. Cystidia present. Spores hyaline, smooth, upon drying becoming angular. In soil or on living or dead wood. Exemplar species: *Stereopsis radicans* (Berk.) D.A. Reid and *Stereopsis globosa* (Hjortstam & Ryvarden) Sjkvist.

### Morphology, Ecology, Life strategy and Distribution

**Figure 5:**
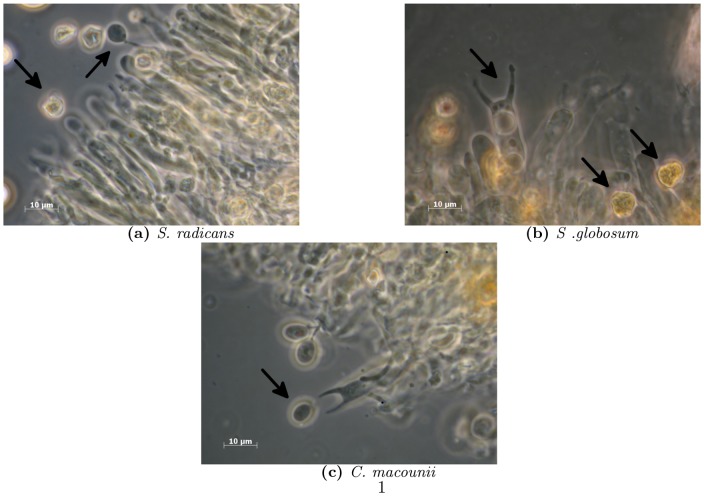
Microscope images of hymenium structures. Hymenium structures in 3% KOH showing basidiospores and palisades of basidia. a) *Stereopsis radicans*, arrows indicating developing spore (subglobose) attached to sterigmata, and mature dried (angular) spore. b) *Stereopsis globosa*, arrows indicating basidia with developed sterigmata, and dried angular spores. c) *Clavulicium macounii* arrow indicating free floating mature spore (cylindrical).

Morphological synapomorphies supporting the higher ranks of agaricomyceteous fungi are often absent and it appears as if many morphological traits are plastic or have evolved convergently in several lineages [3], [20], [21]. The corticioid fruiting body type, as seen in *Clavulicium*, is present in all orders of Agaricomycetes Dowell, but sometimes rare, like in the Phallomycetidae K. Hosaka, Castellano & Spatafora [6], [22]. Stipitate sporocarps with a smooth hymenophore that are characteristic of *Stereopsis*, are also present in Agaricales Underw., Thelephorales Corner ex Oberw. Hymenochaetales Oberw. and Polyporales Gum. Two-spored basidia, an apparent synapomorphy for the *Stereopsis* clade, are present in many other lineages of Agaricomycetes Dowell, e.g., in Atheliales Jlich and Agaricales Underw., but only in a few species. If *Clavulicium macounii* is sister species to the *Stereopsis* clade, a parsimony perspective would lead to the conclusion that the feature of two sterigmata has prevailed since the split between *C. macounii* and the *Stereopsis* clade. This clade would be up to 290 my, based on a comparison of a dated genome phylogeny [10] and the position of Hymenochaetales Oberw. and Auriculariales J. Schrt. The two sterigmata is an indication of an amphithallic reproductive mode [23], [24], where two nuclei are sorted to each spore, often omitting outcrossing. The highly refractive contents of the spores and the way in which the spores become angular and amber-like upon drying in *Stereopsis radicans*, *S. globosa* and *C. macounii* (fig 5a & b), is a morphological feature which separates them from species in other orders of Agaricomycetes Dowell.

*Stereopsis radicans* and *S. globosa* are both found in tropical rain forest and cloud forest. According to [25] *S. radicans* is pantropically distributed, but we have only examined specimens from the neotropics. *Stereopsis globosa* has only been reported from Central and South America. The high molecular diversity observed among the limited number of specimens here referred to the morphological species *S. radicans* and *S. globosa* indicates that a high number of cryptic species may exist.

*Clavulicium macounii* is found on strongly decayed wood, mostly in boreal conifer forests, and like *Stereopsis globosa* it forms effused sporocarps with a smooth hymenophore. The micromorpological characters are the same as those in *S. globosa* and *S. radicans*, with the exception of the spore shape, which in *C. macounii* is ellipsoid (Fig 5).

Both *Stereopsis* and *Clavulicium* sensu lato display a considerable micromorphological diversity, for example in spore morphology, presence or absence of cystidia, and presence or absence of clamps. The specific spore morphology seen in *Stereopsis radicans* and *S. globosa*, are not known from other species of *Stereopsis* or *Clavulicium*. Whether any of those species currently classified as *Stereopsis* or *Clavulicium* that are not yet included in any molecular phylogenetic study could belong to Stereopsidales is hard to predict, there are no obvious candidates. Previous studies have found that *S. vitellina* belongs in Atheliales [7]; *Stereopsis humphreyi* belongs in Agaricales [7], [26]; a previous member of *Clavulicium*, *Membranomyces delectabile*, has been recovered in Cantharellales [3], [5], [21]. However, it is likely that further sampling in the tropics, focusing on corticioid and stipitate stereoid species will yield more species to add to Stereopsidales.

## Materials and Methods

### Taxon sampling

To place the species of study in an order or to verify the need for a new order for them, samples from all orders of Agaricomycetes [1], [2] were included in the dataset by three representatives, where available. GenBank sequences were from three recent molecular studies [2], [6], [27] listed in Table S1 in File S1. In addition, species which might be related to the study group were sought in public archives by using the BLAST algorithm [12], and the ten best matches for each gene were included in the datasets. Information on BLAST hits added to the dataset is listed in Table S2 in File S1.

### PCR and sequencing

We amplified sequences from four nuclear genomic regions: *tef1*, *rpb2*, SSU and LSU. Thirty one sequences were newly generated for this study. For detailed information on the specimens see table 2. PCR amplifications were carried out using PuRe Taq Ready-to-go PCR beads (Amersham Biosciences, Uppsala) following the manufacturer€s recommendations. SSU was amplified using primer pairs NS1/NS4 and NS3/NS8 [28] 40 cycles using standard amplification parameters: initial denaturation in 95 C for 5 sec., and 94 C for 30 s, 60 C annealing temperature 30 s., and 72 C extension for 60 s. The amplification products were checked with electrophoresis for the presence of multiple products. Amplification products were purified using the QIA Quick PCR Purification Kit (Qiagen), following the manufacturers manual. The concentration of products was measured in a RNA/DNA calculator (Pharmacia biotech). Sequencing of the SSU region was performed was performed at Macrogen Incorporating (Korea), using primers pairs NS1, NS2, NS3, NS4, NS8 and NS51 [28]. Sequencing of *tef1*, *rpb2* and LSU were performed as described in [29].

**Table 2:** Collection ID, Collection information and GenBank numbers of newly generated sequences.

Taxon	Voucher (Herbarium/Collection)	Country	*rpb2*	*tef1*	nrDNA
*Clavulicium globosum*	GB/KHL12592	Costa Rica	KC203501	KC203515	KC203495
*Clavulicium globosum*	GB/KHL11228	Costa Rica		KC203513	KC203493
*Clavulicium globosum*	O/LR45201	Belize		KC203509	KC203489
*Clavulicium sp*	O/KHL12754	Costa Rica		KC203510	KC203490
*Clavulicium sp.*	O/KHL12949	Costa Rica		KC203511	KC203491
*Clavulicium cf.globosum*	O/KHL15523	Brazil	KC203504	KC203518	KC203498
*Clavulicium macounii*	GB/KHL12129	Sweden		KC203514	KC203494
*Clavulicium macounii*	GB/B.Nordn37145	Sweden		KC203512	KC203492
*Clavulicium macounii*	GB/KHL15566	Sweden	KC203506	KC203520	KC203500
*Stereopsis cf. radicans*	O/KHL15528	Brazil	KC203503	KC203517	KC203497
*Stereopsis radicans*	O/LR45395	Belize	KC203502	KC203516	KC203496
*Stereopsis radicans*	Cort/Baroni8943	Venezuela		KC203507	KC203487
*Stereopsis radicans*	GB/Ryvarden699	Ecuador		KC203508	KC203488
*Stereopsis sp.*	O/KHL15544	Brazil	KC203505	KC203519	KC203499

### Sequence editing, alignment and phylogenetic analyses

Sequences were assembled in Staden [30] using Pregap4 and Gap4, in Geneious (Geneious version 5.5.6 created by Biomatters available from http://www.geneious.com/) and in Sequencher 4.1 (Gene Codes Ann Arbor, Michigan) One sequence each of *Stereopsis radicans* and *Clavulicium macounii* were blasted using blastn [12] for each genetic marker (*tef1*, *rpb2*, SSU, and LSU, the combined SSU-LSU search only gave SSU hits), the top 10 blast hits for each were aligned to the respective dataset, and sequences that could not be aligned (one occurrence) were thereafter removed.

Sequences were aligned using Mafft [31] with default settings followed by manual inspection in Seaview [32]. *Tef1*and *rpb2* sequences were blasted against RNA reference sequences in NCBI, aligned to two of the reference sequences, the introns removed, and the alignment trimmed at both ends. The *tef1*alignment was adjusted by eye in Seaview for four sequences. SSU was additionally aligned by structure in RNAsalsa [33] (using the structure model for *Coprinus cinereus* reference sequence M92991, found in the European ribosomal database at http://bioinformatics.psb.ugent.be/webtools/rRNA/). Ribosomal genes have complex secondary structures of loops and stems, where the nucleotide in a stem is paired with another nucleotide, whereas nucleotides in a loop are freely evolving. Thus any substitution in a stem will lead to another substitution, in the nucleotide pair. In a study on metazoan datasets [34] it was shown that in some cases structure aligned sequences in combination with a structure model perform better than analyses where secondary structure was not taken into account.

The datasets were analysed separately with Bayesian Markov chain Monte Carlo sampling and Maximum likelihood. Bayesian analyses were conducted in Mr Bayes 3.2.1 [35] using reversible model jump + 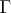, with four parallel runs starting from random trees and sampling one tree every 1000 generations, and running until the standard deviation of split frequencies had stabilized under 0.05. Maximum likelihood was conducted in RaxML [36], [37] using GTR + 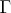, and for the structure aligned SSU, RaxML was called with the consensus structure obtained from RNAsalsa, and Structure model 6A. The alignments and resulting trees are available at http://purl.org/phylo/treebase/phylows/study/TB2:S14833.

### Recombination detection and SH-tests

Recombination tests were conducted in RDP4 [38] for each genetic marker separately. Settings used for RDP4 were as follows: an initial scan with RDP; using external and internal reference; window between 90–100%, Geneconv, Chimera, Maxchi, secondary bootscan and secondary siscan with default settings. Detected recombination events were rechecked with all methods, and the alignments for all recombination events were manually inspected.

We tested whether alternative groupings could be rejected by our data using SH topology tests [11]. These tests compare the log likelihood values of two competing hypotheses under a given p value. In this way it is possible to test whether one tree has a significantly higher likelihood than another. We wanted to know if the results of the individual gene trees for *Stereopsis radicans*, *S. globosa* and *Clavulicium macounii*, could be rejected in the conflicting trees, and whether the *Stereopsis* clade could be included in Polyporales.

SH tests were conducted in RaxML for each of the datasets to test several different hypotheses.

H1. *Stereopsis radicans*, *Clavulicium macounii*, *S. globosa* form a clade. H2. Phallomycetidae, *S. radicans*, *C. macounii*, and *S. globosa* form a clade. H3. Phallomycetidae is monophyletic; *S. radicans*, *C. macounii*, *S. globosa* are monophyletic, thus Phallomycetidae and *Stereopsis* - *Clavulicium* are reciprocally monophyletic. H4. Phallomycetidae and *C. macounii* form a clade. H5. Polyporales and *S. radicans*, *S. globosa* and *C. macounii* form a clade. The best tree for each genetic marker was used as H0, whereas H1, H2, H3, H4 and H5 were tested in turn unless redundant.

### Specimens examined

*Stereopsis radicans*, Surinam, 1879, K(M) 178844 (KEW), TYPE. *Stereopsis radicans*, Venezuela, Aragua st, 30 August 1999, Soil, Baroni8943 (CORT). *Stereopsis radicans*, Ecuador, Napo, Santa Rosa de Quijos, 12 Feb. 1980, On fallen trunk, Montane rain forest alt. ca 1500 m, Ryvarden699 (GB). *Stereopsis radicans*, Belize, Cayo district, Five sisters, Nature trail, 2 November 2002, On dead decidious wood, LR45395(O). *Stereopsis* sp. Brazil, On the ground, KHL15544 (O). *Stereopsis* cf. *radicans*, Brazil, On living tree, KHL 15528 (O). *Stereopsis* cf. *globosum*, Brazil, On living tree, KHL15523 (O). *Clavulicium globosum*, Belize, Cayo district, Blue Hole Nat. Park, Hummingbird trail, 28 October 2002, On dead decidious wood, LR45201 (O). *Clavulicium* sp. Costa Rica, Puntarenas, Coto Brus, Sabalito, Zona Protectora Las Tablas La Neblina, 5 Nov 2004, On angiosperm log, Alt. ca 1350 m, KHL12754 (O). *Clavulicium* sp. Costa Rica, San Jose, Dota, San Gerardo, Sendro la Quebrada, 9 Nov 2004, On strongly decayed wood of angisperm tree, Alt. ca 2400, KHL12949 (O). *Clavulicium globosum*, Costa Rica, Wood, KHL 11228. *Clavulicium* sp. Costa Rica, Puntarenas Coto Brus, Sabalito, Zona Protectora Las Tablas, Progreso, Camino a Cotoncito, 3 November 2004, On wood, Alt. 1560, KHL12592 (GB). *Clavulicium globosum*, Ecuador, Orellana prov, Yasuni National Park, Yasuni Scientific research station, On dead wood, March 2002, Ryvarden 44705, HOLOTYPE. *Clavulicium macounii*, Norway, Nordland, Hemnes, Sjforsen nat. res. Neverbekken, On *Picea abies*, 25 August 2012, KHL 15620 (O). *Clavulicium macounii*, Sweden, Vstergaland, dens par., S of the church, not far from ren, 18 Oct, 2003, *Picea abies* trunk, KHL 12129 (GB). *Clavulicium macounii*, Quebec, Hull, J, Macoun, No 368 Oct, 17 1898 Ex Herb, E, A, Burt, TYPE.

### Nomenclature

The electronic version of this article in Portable Document Format (PDF) in a work with an ISSN or ISBN will represent a published work according to the International Code of Nomenclature for algae, fungi, and plants, and hence the new names contained in the electronic publication of a PLOS ONE article are effectively published under that Code from the electronic edition alone, so there is no longer any need to provide printed copies.

In addition, new names contained in this work have been submitted to MycoBank from where they will be made available to the Global Names Index. The unique MycoBank number can be resolved and the associated information viewed through any standard web browser by appending the MycoBank number contained in this publication to the prefix http://www.mycobank.org/MB/. The online version of this work is archived and available from the following digital repositories:PubMed Central, LOCKSS, GUPEA.

## Supporting Information

File S1 **Tables S1–S2.** Table S1, Genbank numbers of public sequences used in this study. Table S2, Significant BLAST hits.
(PDF)
