# Correction: Stereopsidales - A New Order of Mushroom-Forming Fungi

**DOI:** 10.1371/journal.pone.0106204

**Published:** 2014-08-19

**Authors:** The PLOS ONE Staff

The first author’s name is misspelled in the Taxonomy section of the Discussion. The correct name is: Sjökvist, E.
