# Supplementary material for: Stereopsidales - A New Order of Mushroom-Forming Fungi
Source: PLoS One. 2014 Apr 28;9(4):e95227. doi: 10.1371/journal.pone.0095227 (PMC4002437; doi:10.1371/journal.pone.0095227)
Supplement: File S1 — Tables S1–S2. Table S1, Genbank numbers of public sequences used in this study. Table S2, Significant BLAST hits. (PDF) [file pone.0095227.s001.pdf]

Table S1. Genbank numbers of public sequences used in this study.

Taxon	*rpb2*	*tef*	SSU	LSU
Amylocorticiales				
Amylocorticium cebennense	GU187770	GU187675	GU187612	GU187561
Anomoloma albolutescens	GU187768	GU187671	GU187618	GU187563
Atheliales				
Fibulorhizoctonia sp.	AY885161	AY879115	AY654887	AY635779
Athelia epiphylla	GU187771	GU187676	GU187613	GU187558
Piloderma fallax	GU187797	GU187738	GU187644	GU187535
Leptosporomyces raunkiaerii	GU187791	GU187719	GU187640	GU187588
Auriculariales				
Auricularia sp.	DQ366278	DQ408143	DQ234542	AY634277
Bourdotia sp.		DQ059760	DQ234544	AY635777
Exidia glandulosa		DQ059043	AY293129	AY293179
Boletales				
Serpula himantioides	DQ366283	DQ059046	AF518589	AF518648
Strobilomyces floccopus	AY786065	AY883428	AY662661	AY684155
Suillus pictus	AY786066	AY883429	AY662659	AY684154
Cantharellales				
Clavulina sp.	DQ366286	DQ028589	AY757265	AY745694
Hydnum albomagnum	DQ234553	DQ234568	AY665777	AY700199
Botryobasidium subcoronatum	DQ366284		AY662666	AY647212
Corticiales				
Punctularia strigosozonata	DQ381843	DQ408147	AF518586	AF518642
Vuillemenia comedens	DQ381844		AF518594	AF518666
Dacrymycetales				
Calocera cornea	AY536286	AY881019	AY771610	AY701526
Guepiniopsis buccina	DQ385875	DQ028588	DQ667157	AY745711
Agaricales				
Lepista irina	DQ385885	DQ028591	AY705948	DQ234538
Chondrostereum purpureum	AY218477	DQ457632	AF082851	AF518607
Macrolepiota dolichaula	DQ385886	DQ435785	AY771602	DQ411537
Gloeophyllales				
Gloeophyllum sepiarium	HM536109	HM536110	HM536061	HM536062
Neolentinus lepideus	HM536119	HM536120	HM536076	HM536075
Veluticeps berkeleyi	HM536125	HM536126	HM536082	HM536081
Gomphales				
Gautieria otthii	AY218486	AY883434	AF393043	AF393058

Continued on next page

Table S1 – continued from previous page

Taxon	*rpb2*	*tef*	SSU	LSU
Ramaria rubella	AY786064	AY883435	AY707095	AY645057
Ramaria stricta	DQ219078	DQ219256	-	AF213117
Phaeoclavulina grandis	DQ219074	DQ219252	-	DQ218618
Clavariadelphus truncatus	DQ219064	DQ219240	-	AY574649
Phallales				
Phallus hadriani	DQ408114	DQ435792	AY771601	AY885165
Ileodictyon gracile	DQ219095	DQ219272	-	DQ218635
Protubera canescens	DQ219108	DQ219284		DQ218645
Hysterangiales				
Hysterangium clathroides	DQ218976	DQ219151	-	DQ218547
Trappea pinyonensis	DQ219043	DQ219221	-	DQ218597
Gallacea dingleyae	DQ218959	DQ219137	-	DQ218539
Geastrales				
Sphaerobolus stellatus	DQ219062	DQ219237	-	AF393077
Geastrum floriforme	DQ219049	DQ219227	-	DQ218485
Geastrum pectinatum	DQ219051	DQ219229	-	DQ218602
Hymenochaetales				
Hyphoderma praetermissum	AY787221	AY885150	AY707094	AY700185
Rickenella fibula	DQ408115	DQ435794	AY771599	AY700195
Fomitiporia mediterranea	AY803748	AY885149	AY662664	AY684157
Jaapiales				
Jaapia argillacea	GU187788	GU187711	AF518581	GU187581
Polyoporales				
Donkioporia expansa	HM536102	HM536103	HM536053	HM536052
Fomitopsis pinicola	AY786056	AY885152	AY705967	AY684164
Climacodon septentrionalis	AY780941	AY885151	AY705964	AY684165
Phlebia radiata	AY218502	AY885156	AF026606	AF287885
Russulales				
Hericium americanum	DQ408127	DQ028585	AY665778	DQ411538
Echinodontium tinctorium	AY218482	AY885157	AF026578	AF393056
Lactarius deceptivus	AY803749	AY885158	AY707093	AY631899
Sebacinales				
Piriformospora indica	DQ408131	AJ249911	AY293147	AY293202
Tremellodendron pallidum	DQ408132	DQ029196	AY766081	AY745701
Thelephorales				
Clavariadelphus truncatus	DQ408133	DQ059053	AY752971	AY631900
Polyozellus multiplex	DQ408134	DQ028596	AY771600	AY634275

Continued on next page

Table S1 – continued from previous page

Taxon	*rpb2*	*tef*	SSU	LSU
Trechisporales				
Trechispora alnicola	DQ408135	DQ059052	AY657012	AY635768
Trechispora sp.	DQ408136	DQ059051	AY803753	AY647217

Table S2. Significant BLAST hits.

Accession nr.	*Description*	*E-value*	Max. ident.
tef			
GU187720.1	*Leucogyrophana romellii*	0.0	81%
GU187715.1	*Leucogyrophana lichenic*	0.0	81%
JN164896.1	*Trametes gibbosa*	0.0	81%
DQ521420.1	*Haplotrichum conspersum*	0.0	80%
GU187701.1	*Gyrodon lividus*	0.0	80%
HM467599.1	*Perenniporiella neofulv*	0.0	80%
GU187755.1	*Simocybe serrulata*	0.0	80%
JN164883.1	*Trametes cubensis*	0.0	80%
GU187691.1	*Coniophora prasinoides*	0.0	80%
JN164881.1	*Trametes polyzona*	0.0	80%
DQ059047.1	*Phyllotopsis sp.*	0.0	81%
AY883429.1	*Suillus pictus*	0.0	80%
DQ028603.1	*Trametes versicolor*	0.0	81%
AY879116.1	*Boletellus projectellus*	0.0	79%
AY885152.1	*Fomitopsis pinicola*	0.0	80%
JN164878.1	*Trametes versicolor*	0.0	83%
JN164888.1	*Trametes ectypa*	0.0	83%
JN164892.1	*Trametes villosa*	0.0	83%
JN164891.1	*Trametes hirsuta*	0.0	83%
JN164889.1	*Trametes pubescens*	0.0	83%
SSU			
DQ834916.1	*Resinicium saccharicola*	0.0	95%
AF026615.1	*Resinicium bicolor*	0.0	95%
EU888830.1	*Pycnoporus sp.*	0.0	95%
DQ437679.1	*Alloclavaria purpurea*	0.0	94%
GU182936.1	*Pycnoporus sp.*	0.0	94%
DQ834915.1	*Resinicium friabile*	0.0	95%
AY336774.1	*Antrodiella semisupina*	0.0	94%
AY336751.1	*Trametes versicolor*	0.0	94%
AY707094.1	*Hyphoderma praetermissum*	0.0	94%
AY309019.1	*Trametes versicolor*	0.0	94%
DQ873636.1	*Mycoacia pinicola*	0.0	98%
DQ873594.1	*Globulicium hiemale*	0.0	98%

Continued on next page

Table S2 – continued from previous page

Accession	Description	E-value	Max. ident.
AY752973.1	*Cyphellostereum laeve*	0.0	98%
AY771599.1	*Rickenella fibula*	0.0	97%
DQ437679.1	*Alloclavaria purpurea*	0.0	97%
DQ917659.1	*Thanatephorus cucumeris*	0.0	97%
AY757266.1	*Ceratobasidium sp.*	0.0	97%
AY946268.1	*Thanatephorus cucumeris*	0.0	97%
EF025042.1	*Uncultured eukaryote*	0.0	97%
EF023749.1	*Uncultured Auriculariaceae*	0.0	97%
LSU			
JN710593.1	*Steccherinum sp.*	0.0	88%
JN710592.1	*Steccherinum sp.*	0.0	88%
JN710560.1	*Junghuhnia nitida*	0.0	88%
DQ911614.1	*Auriscalpium vulgare*	0.0	88%
JN710556.1	*Junghuhnia japonica*	0.0	88%
JN710533.1	*Flabellophora sp.*	0.0	88%
AY586656.1	*Gloeoporus taxicola*	0.0	88%
JN939577.1	*Hypochnicium wakefieldiae*	0.0	88%
AF506471.1	*Scytinostromella hetero*	0.0	88%
JN710576.1	*Nigroporus vinosus*	0.0	88%
DQ873631.1	*Hyphodontia subalutacea*	0.0	94%
EU118621.1	*Cyphellostereum laeve*	0.0	94%
JQ700296.1	*Daedalea quercina*	0.0	93%
JN710586.1	*Steccherinum cf.*	0.0	93%
AF506385.1	*Creolophus cirrhatus*	0.0	93%
JN710588.1	*Steccherinum murashkinskyi*	0.0	93%
JQ700297.1	*Piptoporus betulinus*	0.0	93%
AY629318.1	*Grifola frondosa*	0.0	94%
JN710562.1	*Junghuhnia rhinocephala*	0.0	93%
AF506459.1	*Hericium coralloides*	0.0	93%
rpb2			
GU187810.1	*Suillus bresadolae*	2e-176	77%
DQ385878.1	*Bolbitius vitellinus*	3e-160	77%
AY786059.1	*Hygrophoropsis aurantiaca*	1e-159	76%
DQ472716.1	*Gymnopus contrarius*	2e-157	77%
DQ859892.1	*Sarcomyxa serotina*	1e-153	76%
AY786063.1	*Pluteus romellii*	6e-137	75%
JN164850.1	*Trametes versicolor*	4e-129	75%
JN164859.1	*Trametes gibbosa*	1e-109	75%
AY536286.1	*Calocera cornea*	1e-108	75%
HM536123.1	*Veluticeps abietina*	6e-107	75%
DQ056143.1	*Coprinellus disseminatus*	0.0	75%
DQ472730.1	*Kuehneromyces rostratus*	0.0	75%
GU187767.1	*Athelia sp.*	0.0	75%
AY786058.1	*Grifola sordulenta*	0.0	74%

Continued on next page

Table S2 – continued from previous page

Accession	Description	E-value	Max. ident.
AY218482.2	*Echinodontium tinctorium*	3e-177	73%
HM536107.1	*Gloeophyllum protractum*	2e-124	77%
GU384651.1	*Entoloma vinaceum*	3e-112	75%
JN710739.1	*Steccherinum tenue*	5e-110	76%
DQ219075.1	*Phaeoclavulina sp.*	9e-103	76%
DQ381840.1	*Tulasnella sp.*	9e-103	74%
